# Supplementary figures and images for: Survival of hibernating little brown bats that are unaffected by white-nose syndrome: Using thermal cameras to understand arousal behavior
Source: PLoS One. 2024 Feb 6;19(2):e0297871. doi: 10.1371/journal.pone.0297871 (PMC10846716; doi:10.1371/journal.pone.0297871)

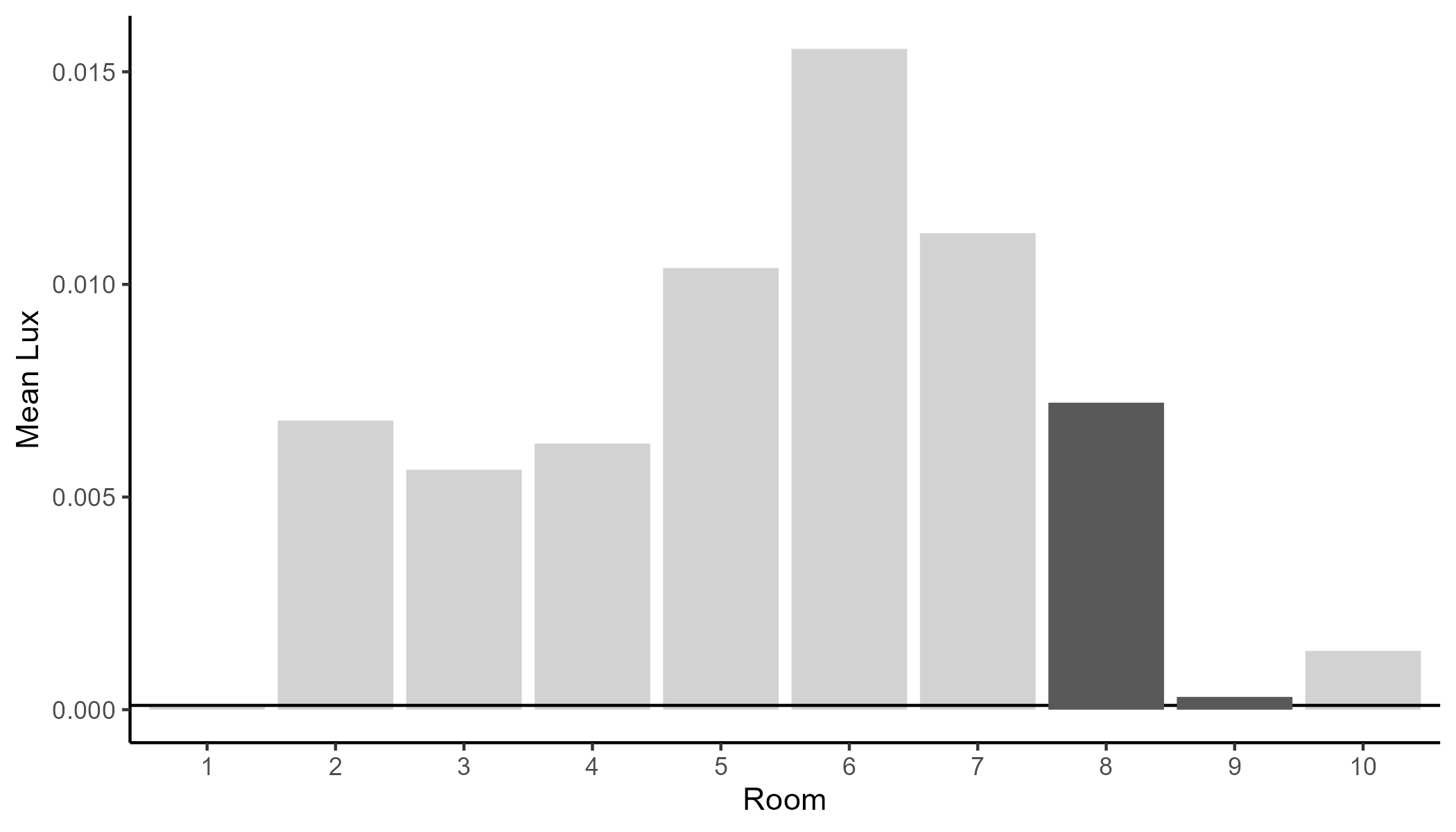

Supplement: S1 Fig — Video was collected from rooms 8 and 9 (dark grey). (TIF) [file pone.0297871.s001.tif]

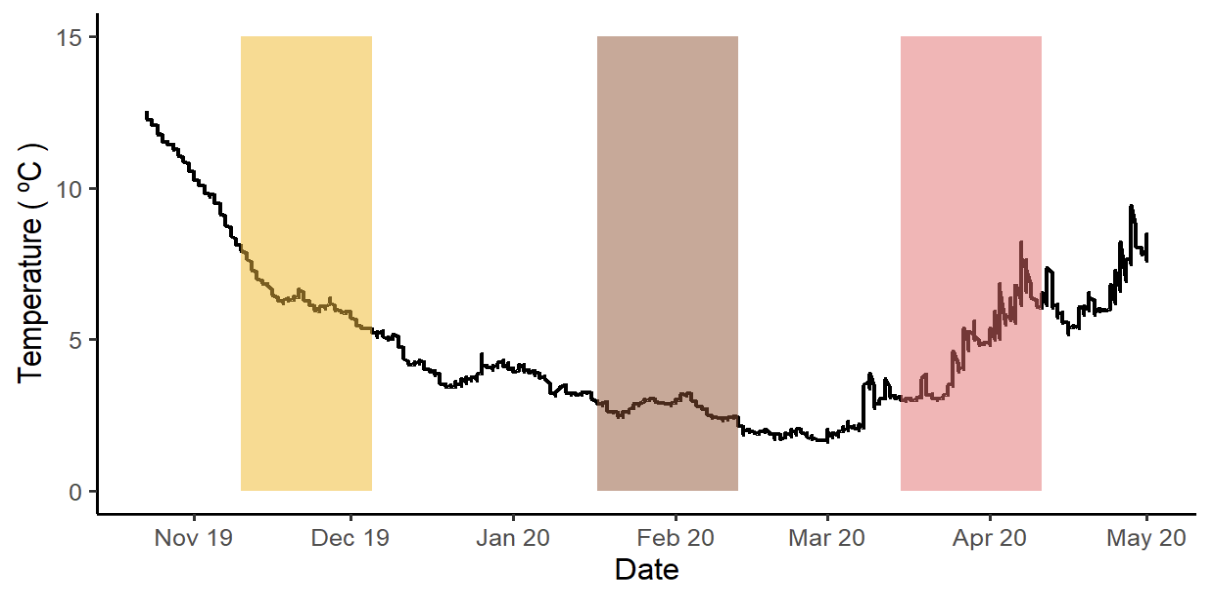

Supplement: S2 Fig — The early (yellow), middle (brown), and late (pink) hibernation periods used in this study are shown via the colored rectangles. Data courtesy of B. A. Daly. (TIF) [file pone.0297871.s002.tif]
