# Supplementary material for: Survival of hibernating little brown bats that are unaffected by white-nose syndrome: Using thermal cameras to understand arousal behavior
Source: PLoS One. 2024 Feb 6;19(2):e0297871. doi: 10.1371/journal.pone.0297871 (PMC10846716; doi:10.1371/journal.pone.0297871)
Supplement: S1 File — (DOCX) [file pone.0297871.s003.docx]

**Supplement**

**Study Site**

Tippy Dam is a hydroelectric dam located near Wellston, Manistee County, Michigan (44.26°N, 85.94°W) that was built in 1918. Manistee County is covered in glacial till [1], and other potential hibernacula, such as caves and cracks or crevices in the bedrock, are not available in this area. The dam consists of a concrete spillway, a power house, and earthen embankments on both sides. Inside, the spillway consists of 10 rooms, with rooms 2–10 connected by an elevated walkway, about 8 m above the floor. Bats roost mostly on the internal walls between chambers in small-to-large groups [2]. There are ventilation holes in rooms 2–10, allowing bats and light to enter, and although the spillway is dimly lit, enough daylight penetrates for humans to walk safely without a flashlight. The site is well protected from disturbance, and no humans are allowed to enter the spillway during the hibernation period, except in emergencies [3].

The temperature within the spillway fluctuates considerably during the hibernation season, because the air temperature inside is controlled by the temperature of the water behind the dam [2]. During winter 2019­­­­–2020, as part of another study, temperatures were measured by B. A. Daly (in litt.) near the ceiling, in a small “window,” between rooms 8 and 9. Maximum temperatures during hibernation occurred in October, with a sharp decline through December, steadier temperatures from January to early March, and then an increase beginning in mid-March. Internally, ambient temperatures ranged between 1 and 13°C, and relative humidity exceeded 90% for most of the hibernating period (B. A. Daly, in litt.).

**Video Monitoring**

Bats were monitored using thermal-imaging surveillance cameras (Model Q1942-E, Axis Communications, Lund, Sweden). One camera was set in room 8 and another in room 9 of the spillway, with each camera facing the wall separating the two chambers. The color setting for each camera was “Fire and Ice,” and the resolution was 800 by 600 pixels at 30 frames/sec. Each unit was connected to a single network video recorder with 4 Tb of storage (Model D2000, Axis Communications), which continuously logged footage over 174 days, from 23 October 2019 to 15 April 2020. Uninterrupted power was supplied to the cameras and recorder through an extension cord, snaked into the spillway through one of the ventilation openings from an external outlet near the power house. Although other studies (e.g., Hayman et al., 2017) encountered problems with rodents chewing through electrical cords, no animals other than bats and humans are capable of accessing the spillway at Tippy Dam, and the units never lost power during the 6-month study. Videos were stored and analyzed as Advanced Systems Format (ASF) files (Microsoft Corporation, Redmond, Washington), which are designed to store audio and visual information for later streaming.

**Circular Statistics**

All mean directions (arousal times) are reported in decimal hours. For the overall hibernation season (172 days), mean arousal time was 1:01AM (mean = 1.02, *ρ* = 0.35, p < 0.001). Mean arousal time was 12:42AM for the early period (mean = 0.71, *ρ* = 0.25, p < 0.001), 12:23AM for the middle period (mean = 0.38, *ρ* = 0.33, p < 0.001), and 1:55AM for the late period (mean = 1.92, *ρ* = 0.48, p < 0.001). Arousal “hour” is defined as 60 min from the start of an hour until the end of that hour; e.g., if the peak arousal hour is stated as 8:00PM, the hour is defined as occurring between 8:00 and 8:59PM. Peak arousal hour, or the hour with the greatest aggregate number of aroused bats, was 1:00AM for the overall hibernation season, 8:00PM in the early period, 11:00PM in the middle interval, and 1:00AM for the late period (Figure S4).

**Social thermoregulation and clustering of aroused animals**

Means reported here are ± 95% CI (*n*). Mean cluster size of aroused bats was 10.6 ± 0.03 (*n* = 112,957) for the overall season. Average cluster sizes were 10.0 ± 0.07 (*n* = 15,348), 11.2 ± 0.09 (*n* = 14,156), and 10.6 ± 0.01 (*n* = 20,787) for early, middle, and late periods, respectively. Maximum cluster size of aroused bats was 41 animals.

Only 14.8% of all arousal events were considered simultaneously aroused clusters of 5 or more bats (85.2% considered solitary arousals; Table S1). However, mean number of bats in an aroused cluster was 10.6 bats. Consequently, for every 100 arousal events, 85.2 involved solitary animals, but 14.8 events included a total of 156.9 individuals (14.8 events * 10.6 bats) that were part of a cluster of 5 or more arousing bats; hence, 68.3% of the animals (156.9 of the total 242 bats in 100 arousal events) aroused as part of a cluster.

**Table S1**. Percentage of arousals considered clusters, maximum number of bats simultaneously aroused in one frame, and mean number of bats simultaneously aroused in one frame, for each hibernation period in both rooms. Means are ± 95% CI (n). An aroused cluster was defined as 5 or more aroused bats in contact with each other.

| Period | Percent of arousals considered clusters | Maximum number of bats simultaneously aroused per frame | Mean number of bats simultaneously aroused per frame |
| --- | --- | --- | --- |
| Early | 14.8% | 124 | 125.6 ±  3.6 (2,016) |
| Middle | 14.4% | 134 | 125.6 ±  2.4 (7,704) |
| Late | 15.9% | 339 | 212.3 ±  8.7 (2,301) |
| Overall | 14.8% | 339 | 142.2 ±  2.4 (12,021) |

Maximum number of simultaneously aroused bats anywhere in a single frame was 339 (Table S1). The largest number of simultaneously aroused bats in each timeframe was 133 in the early period, 159 in the middle, and 339 in the late period. Mean number of simultaneously aroused bats was 142.2 ± 2.4 (*n* = 12,021) for the overall season (Table S1).

**References**

1. Albert DA, Denton SR, Barnes BV. Regional Landscape Ecosystems of Michigan. University of Michigan School of Natural Resources, Ann Arbor, MI; 1986.
2. Kurta A, Caryl J, Lipps T. Bats and Tippy Dam: species composition, seasonal use, and environmental parameters. *Mich Acad* 1997;473–490.
3. Kurta A, Teramino JA. A Novel Hibernaculum and Noteworthy Records of the Indiana Bat and Eastern Pipistrelle (Chiroptera: Vespertilionidae). *Am Midl Nat* 1994;132(2), 410–413.
